# Evaluation of DNA barcodes in *Codonopsis* (Campanulaceae) and in some large angiosperm plant genera

**DOI:** 10.1371/journal.pone.0170286

**Published:** 2017-02-09

**Authors:** De-Yi Wang, Qiang Wang, Ying-Li Wang, Xiao-Guo Xiang, Lu-Qi Huang, Xiao-Hua Jin

**Affiliations:** 1 State Key Laboratory of Systematic and Evolutionary Botany, Institute of Botany, Chinese Academy of Sciences, Beijing, China; 2 University of Chinese Academy of Sciences, Beijing, China; 3 Baotou Medical College, Donghe District, Baotou City, Inner Mongolia, China; 4 National Resource Centre for Chinese Materia Medica, China Academy of Chinese Medical Science, Beijing, China; 5 Southeast Asia Biodiversity Research Institute, Chinese Academy of Science (CAS-SEABRI), Menglun, Mengla, Yunnan, China; The National Orchid Conservation Center of China; The Orchid Conservation & Research Center of Shenzhen, CHINA

## Abstract

DNA barcoding is expected to be one of the most promising tools in biological taxonomy. However, there have been no agreements on which core barcode should be used in plants, especially in species-rich genera with wide geographical distributions. To evaluate their discriminatory power in large genera, four of the most widely used DNA barcodes, including three plastid regions (*matK*, *rbcL*, *trnH-psbA*) and nuclear internal transcribed spacer (nrITS), were tested in seven species-rich genera (*Ficus*, *Pedicularis*, *Rhodiola*, *Rhododendron*,*Viburnum*, *Dendrobium* and *Lysimachia*) and a moderate size genus, *Codonopsis*. All of the sequences from the aforementioned seven large genera were downloaded from NCBI. The related barcodes for *Codonopsis* were newly generated in this study. Genetics distances, DNA barcoding gaps and phylogenetic trees of the four single barcodes and their combinations were calculated and compared in the seven genera. As for single barcode, nrITS has the most variable sites, the clearest intra- and inter-specific divergences and the highest discrimination rates in the seven genera. Among the combinations of barcodes, ITS+*matK* performed better than all the single barcodes in most cases and even the three- and four-loci combinations in the seven genera. Therefore, we recommend ITS+*matK* as the core barcodes for large plant genera.

## Introduction

DNA barcoding, the use of a short gene sequence from a standardized region of the genome as a tool for species identification, provides new tools for use in biological taxonomy [1–5]. It has shown promise in providing a practical, standardized, species-level identification tool that can be used for taxonomic research, population genetics [6], phylogenetics [7], biodiversity assessment [8], and ecological studies [9–11]. An ideal DNA barcode should be variable enough to resolve closely related species and short enough for easy experimental manipulation at low cost [12]. The cytochrome oxidase I (COI) for the zoological community appears to generally fulfill these criteria [11, 13–15]. In contrast, there is no universally accepted counterpart barcode for plants yet [16]. In the past decade, four loci widely used in plant molecular systematics, namely, ITS, *matK*, *rbcL* and *trnH-psbA*, have been extensively evaluated [3, 16–19]. The lack of resolving power for single barcodes has led to the transition from a single- to a multi-region barcoding system [3, 4, 20–24]. Specifically, the combined use of short segments of the chloroplast genes *matK* and *rbcL* was proposed by the Plant Working Group of the Consortium for Barcodes of Life [3, 14].

Despite the significant progress made in the DNA barcoding of higher plants, some obstacles still hinder its extensive application in plant taxonomy [8]. Firstly, rates of successful amplification and sequencing of candidate DNA makers are highly variable among plant taxa [23, 25]. Secondly, discriminating closely related or recently evolved species remains a challenge for DNA barcoding [8, 14, 26]. Furthermore, one of the biggest challenges is the lack of a broad sampling of well-dispersed species across all plant taxa. It has been proven that barcoding based solely on a limited number of DNA sequences was often inappropriate at the species-specific level [15, 26]. Finally, to date, species discrimination within a genus has been evaluated in a number of cases; however, the barcoding of species-rich genera is particularly difficult and has still not been sufficiently evaluated [21]. Recently, several studies of large plant genera [21, 23, 27–29], such as *Pedicularis* (328 samples representing 88 species), *Rhododendron* (531 samples representing 173 species), and *Dendrobium* (1698 accessions representing of 184 species), have been conducted. Particularly, based on the comparative analyses of barcodes in five plant genera with range from large to moderate size (*Dendrobium*, *Ficus*, *Lysimachia*, *Paphiopedilum*, *Pedicularis*), Xu et al. [21] proposed the combination of *matK* + ITS as the core barcode for large flowering plant genera.

*Codonopsis* s.l. (Campanulaceae) consists of 42 species, mainly distributed in Central, East and South Asia [5, 30, 31]. Many *Codonopsis* species are widely used in traditional medicine and foods across their distributed regions. Fresh or dried caudices and roots of *Codonopsis pilosula* and *C*. *tangshen* have a long history of use as herbal medicines "Dangshen" in China [5, 30]. The roots and caudices of other *Codonopsis* species, including *C*. *tubulosa*, *C*. *subglobosa*, *C*. *clematidea* and *C*. *lanceolata*, are used as vegetables across several Asian countries [5, 32]. Because of the highly similar morphological appearance of the roots and caudices of *Codonopsis* species, DNA barcodes may be valuable for the accurately identifying *Codonopsis* material. According to some studies, *Codonopsis* is a very difficult genus to identify due to its rich and complex species composition, dynamic evolutionary history, and extensive plastid genome rearrangements during diversification [30, 31, 33]. Therefore, the genus *Codonopsis* provides an excellent opportunity to evaluate the application of DNA barcoding.

In this study, our aims were as follows: (1) to evaluate the performance of DNA barcodes in *Codonopsis*; and (2) the performance of barcodes for species identification in large plant genera.

## Materials and methods

### Plant materials, DNA extraction, PCR amplification, sequencing and sequence downloaded

A total of 140 individuals of 35 *Codonopsis* species were used in this study (Table A in S1 File). In the present study, healthy and fresh leaves of each plant were collected and dried immediately in silica gel for DNA extraction. Total genomic DNA was isolated from approximately 1 g of dried leaves following a modified cetyltrime-thylammonium bromide (CTAB) protocol. Three plastid barcodes (the coding genes *matK* and *rbcL*, and the spacer *trnH-psbA*) and a nuclear internal transcribed spacer (nrITS) were amplified and sequenced using universal primers (Table B in S1 File). Polymerase chain reaction (PCR) was used to amplify the selected DNA regions. The PCR mixture (25 μL) contained approximately 10 ng (1–2 μL) of template DNA, 12.5 μL of 2×PCR mix (0.005 units/μL Taq DNA polymerase; 4 mM MgCl_2_; and 0.4 mM dNTPs), 0.2 μL of each primer and 6.5–7.5 μL of ddH_2_O. The conditions of PCR were following Raskoti et al [34]. The sequencing reactions were performed using the Applied Biosystems Prism Bigdye Terminator Cycle Sequencing Kit (FosterCity, CA).

Large genera (here considered as about 100 species or more for each genus) were chosen based on a literature survey using Web of Sciences (Accessed by Jan. 12, 2016). Seven genera, including *Dendrobium*, *Ficus*, *Lysimachia*, *Pedicularis*, *Rhodiola*, *Rhododendron*, and *Viburnum*, were chosen based on the number of species and the barcodes used [21, 23, 27, 28, 35–37]. Most of these genera have more than 200 species. Because the DNA barcoding results of *Dendrobium* [21] and *Lysimachia* [37] from prior studies could be used directly for comparison, here we focused on the other five genera. All sequences for the four extensively used barcodes (ITS, *matK*, *rbcL* and *trnH-psbA*) of the five species-rich genera were downloaded from NCBI. In order to improve accuracy of the evaluation of barcodes, the downloaded sequences were filtered and omitted if they met the following criteria: i) had a length less than 300 bp (length relative to ITS2, which has a length about 300 bp); ii) were with low quality (such as N/? in sequence); iii) lacked a voucher or were only identified to genera (voucher is important for the reliability of sequence). To save computational time, the representatives of each species were limited to fifteen samples. Conclusively, the number of species and individuals in these five genera are 858 sequences of 63 species in *Ficus*, 1306 sequences of 88 species in *Pedicularis*, 672 sequences of 47 species in *Rhodioda*, 1540 sequences of 130 species in *Rhododendron*, 694 sequences of 56 species in *Viburnum*. Although there are studies of the DNA barcoding on other three large genera, i.e., *Begonia* [38] and *Astragalus* [39], *Paphiopedilum* [40], very few species or different sampling strategy and/or barcodes had been included in the analyses. For example, there are about 3000 species in *Astragalus* [41], only eight species were included in the DNA barcoding research [39]. Therefore, these three genera were excluded from our further analyses. The taxa and GenBank accession numbers used in this study are shown in Tables C-G (see S1 File).

### Sequence alignment, genetic distance and barcoding gap

DNA barcodes were aligned with Clustal X V2.0 [42] and manually adjusted in BioEdit v7.2.5 [43]. Then, SequenceMatrix 1.7.7 [44] was used to combine matrixes of single marker into matrixes of multi-makers. The genetic distance of ITS (I), *matK* (M), *rbcL* (R) and *trnH-psbA* (T) and their combinations (I+M, I+R, I+T, M+R, M+T, R+T, I+M+R, I+M+T, I+R+T, M+R+T, I+M+R+T) were systematically analyzed and compared. The output data from BioEdit was processed to calculate the pairwise distance, between group distance and within group distance under the Kimura-2-Parameter (K2P) distances model [45] for each region using MEGA v6.0 [46]. Differences between intra- and inter-specific distances for each pair of four single barcodes were compared using IBM SPSS Statistics v19.0 with Wilcoxon signed-rank tests [47]. To compare barcoding gaps, the distributions of the pairwise intra- and inter-specific distances for each candidate barcode with 0.005 distance intervals were estimated in TaxonDNA with a ‘pairwise summary’ function [48].

### Species discrimination efficiency

The species discrimination efficiency of both single barcodes and their combinations was evaluated by two methods as described below. Firstly, 'Best Match' and 'Best Close Match' functions in TaxonDNA were applied to calculate the accuracy of the barcode regions for species identification. To further evaluate the efficiency of candidate barcodes, a tree-based analysis was conducted to assess the monophyly of individuals representing the same species. The neighbor-joining (NJ) and the unweighted pair group method with arithmetic mean (UPGMA) trees were reconstructed by MEGA v6.0 with the K2P model, and node support was assessed by a bootstrap test with 1000 pseudo-replicates with the K2P distance options [49].

## Results

### Analyses of sequence characteristics

The sequence characteristics of the four regions (ITS, *matK*, *rbcL* and *trnH-psbA*) in *Codonopsis* were shown in Table I in S1 File. The ITS had the highest percentage of variable sites (52.19%), whereas *rbcL* had the lowest (20.50%). *trnH-psbA* had the highest informative sites (30.85%), closely followed by ITS (30.05%) and *rbcL* had the lowest (11.71%).

The sequence characteristics of the four regions in the other five genera (*Ficus*, *Pedicularis*, *Rhodiola*, *Rhododendron*, and *Viburnum*) are summarized in Table J in S1 File. Among the four single barcodes in *Ficus*, the *trnH-psbA* matrix showed the shortest length for the aligned sequences, and ITS provided the highest percentage of variable sites (38.63%) and the highest percentage of informative sites (32.64%), with *rbcL* having the lowest percentages of variable and informative sites (1.79% and 1.30%, respectively).

In *Pedicularis*, length variation exits in *matK* (728-788bp) and in *trnH-psbA* (388-882bp), whereas ITS (662bp) and *rbcL* (606bp) lengths were stable. The *trnH-psbA* provided the highest percentages of variable (67.35%) and informative (58.96%) sites, followed by those of ITS (54.59% and 48.87%, respectively) and *matK* (38.54% and 32.24%, respectively), with *rbcL* having the lowest percentages (14.69% and 14.03%, respectively).

In *Rhodiola*, the *rbcL* matrix had the longest length (1100 bp) of the aligned sequences, and the other matrixes exhibited variable lengths (630–671 bp for ITS, 726–737 bp for *matK*, and 366–381 bp for *trnH-psbA*). ITS had the highest percentages of variable and informative sites (39.94% and 31.59%, respectively).

In *Rhododendron*, the *trnH-psbA* matrix showed the shortest length (515 bp) followed by 701 bp for *rbcL*, 723 bp for ITS, and 765 bp for *matK*. *TrnH-psbA* also had the highest percentages of variable (21.17%) and informative (19.22%) sites followed by *matK* (12.42% and 11.11%, respectively).

In *Viburnum*, the barcode regions varied in length, among which *trnH-psbA* had the lowest range (405–471 bp). ITS had the highest percentages of variable and informative sites were the highest for ITS (29.98% and 23.82%, respectively), whereas *rbcL* had the lowest percentages of variable and informative sites (4.48% and 2.65%, respectively).

### DNA barcode intra- and inter-specific divergence

The mean intra- and inter-specific divergence of candidate barcodes and their combinations were different in the six large genera (Tables J, K in S1 File). Among the single barcodes, our results showed that ITS exhibited the highest mean inter-specific and lower intra-specific divergence in *Ficus*, *Rhodiola*, and *Viburnum*, whereas in *Codonopsis*, *Pedicularis* and *Rhododendron*, *trnH-psbA* had the highest mean inter-specific and lower intra-specific divergence, closely followed by ITS. To further test whether such barcoding gaps exist, the distribution of divergences of each barcodes for the six genera were drawn (Figure A-F in S2 File). Among the single barcodes, there was a clear separation for ITS in the six genera. For the combinations of single barcodes, I+M, I+R, and I+T had less overlap of inter-specific and intra-specific divergence and performed better than the other combinations among the six genera.

The results of the 'Best match' and 'Best close match' analyses indicated that the 'Best match' always performed better than the latter or resulted in equal individual identification rates (Table L in S1 File). All the six genera have varied individual identification rates by the 'Best match' ranging from 37.31% to 84.68% in *Codonopsis*, 2.73%–85.48% in *Ficus*, 51.07%–89.38% in *Pedicularis*, 19.04%–79.16% in *Rhodiola*, 11.94%–49.09% in *Rhododendron*, and 3.15%–67.53% in *Viburnum*. As a whole, the ability of DNA barcodes to discriminate between species was rather high in the six large genera with the exception of *Rhododendron*.

Among the single barcodes, ITS showed the highest individual identification rates among *Codonopsis* (62.07%), *Ficus* (77.88%), *Pedicularis* (83.79%), *Rhodiola* (68.45%), and *Viburnum* (45.34%) using the 'Best Match'; *matK* exhibited a higher discrimination rate in *Codonopsis* (61.65%) and *Pedicularis* (68.82%). Overall, all the single barcodes produced particularly low discrimination rates in *Rhododendron* with the highest rate (28.26% for *trnH-psbA*) generated by the 'Best Match'.

The 'Best Match' analyses of the combined barcodes indicated that they performed differently in the six genera (Table L in S1 File). In *Codonopsis*, I+M showed the highest individual identification rate (82.20%) among two-locus combinations, which was slightly lower than the rate from a four-loci combination (84.68% for I+M+R+T (ITS+*matK*+*rbcL*+*trnH-psbA*)). Furthermore, I+M also exhibited the highest individual identification rate (85.48%) among all the combined barcodes in *Ficus*. In *Pedicularis*, the highest discrimination was 89.80% for I+M+T (ITS+*matK*+ *trnH-psbA*), followed by 89.38% for I+M+R+T; the two loci barcodes I+M and I+T also performed well with rather high discrimination rates of 85.98% and 88.99%, respectively. In *Rhodiola*, the highest discrimination rate was 79.16% for both I+M+T and I+M+R+T; and the two loci barcodes I+M and I+T also performed well with discrimination rates of 76.19% and 77.38%, respectively. In *Rhododendron*, all the combined barcodes had low discrimination rates that were below 50%, and among which I+M+T had the highest (49.09%). In *Viburnum*, I+M+R+T showed the highest discrimination rate (67.53%), slightly more than that of I+R+T (67.05%).

### Tree-based method analyses

Our results indicate that the UPGMA tree provided the better indications of discriminatory power than the NJ tree (Table M in S1 File, Figure A-Z, a-d in S3 File, Figure A-Z, a-d in S4 File, Figure A-Z, a-d in S5 File, Figure A-Z, a-d in S6 File, Figure A-Z, a-d in S7 File, Figure A-Z, a-d in S8 File). The discrimination rate using two phylogenetic methods was high in the six genera with exception *Rhododendron*, for which the discrimination rates were below 50%. All the single barcodes showed lower discrimination rates that were below 50% in *Codonopsis*, among which *rbcL* had the lowest identification rate, and the other three barcodes (ITS, *matK*, and *trnH-psbA*) did not have distinctive discriminatory power with either the NJ and UPGMA tree. Among the other five genera, ITS had the highest discriminatory power with both the NJ tree and UPGMA tree, respectively, with discrimination rates of 68.85% and 72.13% in *Ficus*, 79.55% and 72.73% in *Pedicularis*, 53.19% and 51.06% in *Rhodiola*, and 42.22% and 40.00% in *Viburnum*. Additionally, *matK* had the highest discriminatory power (21.54% and 22.31%) among the single barcodes in *Rhododendron* using both phylogenetic methods.

Among the combined markers in *Codonopsis*, I+M and I+M+R+T showed the highest discrimination rate (66.67%) with both the NJ and UPGMA tree methods. Furthermore, I+M exhibited the highest discriminatory power with both the NJ tree and UPGMA trees, respectively, with discrimination rates of 74.58% and 69.49% in *Ficus*, and 83.91% and 82.76% in *Pedicularis*, respectively. In *Rhodiola*, I+T had the highest discriminatory power (63.83% and 57.45%) with both the NJ tree and UPGMA tree methods, followed by 57.45% and 55.32% for I+M. In *Viburnum*, I+M+R provided the highest discrimination rates with values of 62.07% with the NJ tree and 48.28% with the UPGMA tree. Using combinations of barcodes, however, failed to improve the discriminatory power in *Rhododendron* merely with the highest values being 35.38% and 38.46% for the NJ tree UPGMA tree of I+M+R+T, respectively.

## Discussion

### Evaluation of barcodes for *Codonopsis* (Campanulaceae)

**Fig 1:**
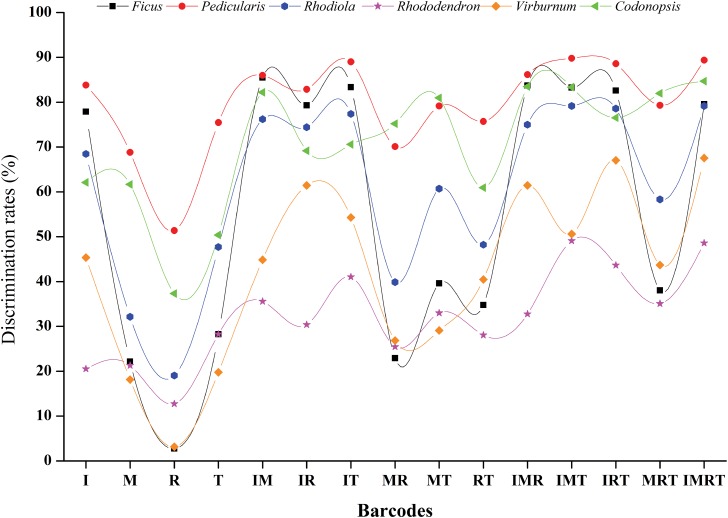
Discremination rates of DNA barcoding in six genera in the analyses of 'Best Match' in TaxonDNA. The lables in X-axis representing all the single barcodes and their combinations used in this study. I: ITS, M: *matK*, R: *rbcL*, T: *trnH-psbA*. IM: ITS + *matK*; IR, ITS + *rbcL*; IT, ITS +*trnH-psbA*;MR, *matK* + *rbcL*; MT, *matK* + *trnH-psbA*; RT, *rbcL* + *trnH-psbA*; IMR, ITS + *matK* + *rbcL*; IMT, ITS + *matK* + *trnH-psbA*; IRT, ITS + *rbcL* + *trnH-psbA*; MRT, *matK* + *rbcL* + *trnH-psbA*; IMRT, ITS + *matK* + *rbcL* + *trnH-psbA*.

Ideally, DNA barcodes should meet several critical criteria: (1) having high inter-specific but low intra-specific divergence so that they can be discriminated from one another; (2) having highly conserved flanking sites for developing universal primers; (3) having appropriately short length for DNA extraction, PCR amplification and sequencing; (4) easy alignment without manual editing [3, 9, 28, 50]. Although the use of DNA barcoding for identification and taxonomy has been controversial [16], a growing number of barcodes have been proposed for plant species identification. A list of barcodes have been proposed as universal barcodes for land plants, such as *rbcL* (easy to be sequenced and aligned in plants) [3]; *matK* (one of the most rapidly evolving plastid coding regions) [3, 16]; ITS (more variable sites and greater intra- and inter-specific divergences) [23, 28, 35, 51, 52]; *trnH-psbA* (variable sites to discriminate recently evolved species) [29, 53, 54]; the *trnL* intron [55] and ycf1 [12], etc. In our study, *rbcL* contained the lowest percentages of informative and variable sites and the lowest discrimination ability among all studied genera (Fig 1). In contrast, ITS contained the highest percentage of variable sites, had greater intra- and inter-specific divergence, the highest discrimination rates and suitable alignment lengths in our study (Fig 1). Given its superior performance, ITS is considered to be an optional core barcode for species-level barcoding *Codonopsis*.

**Fig 2:**
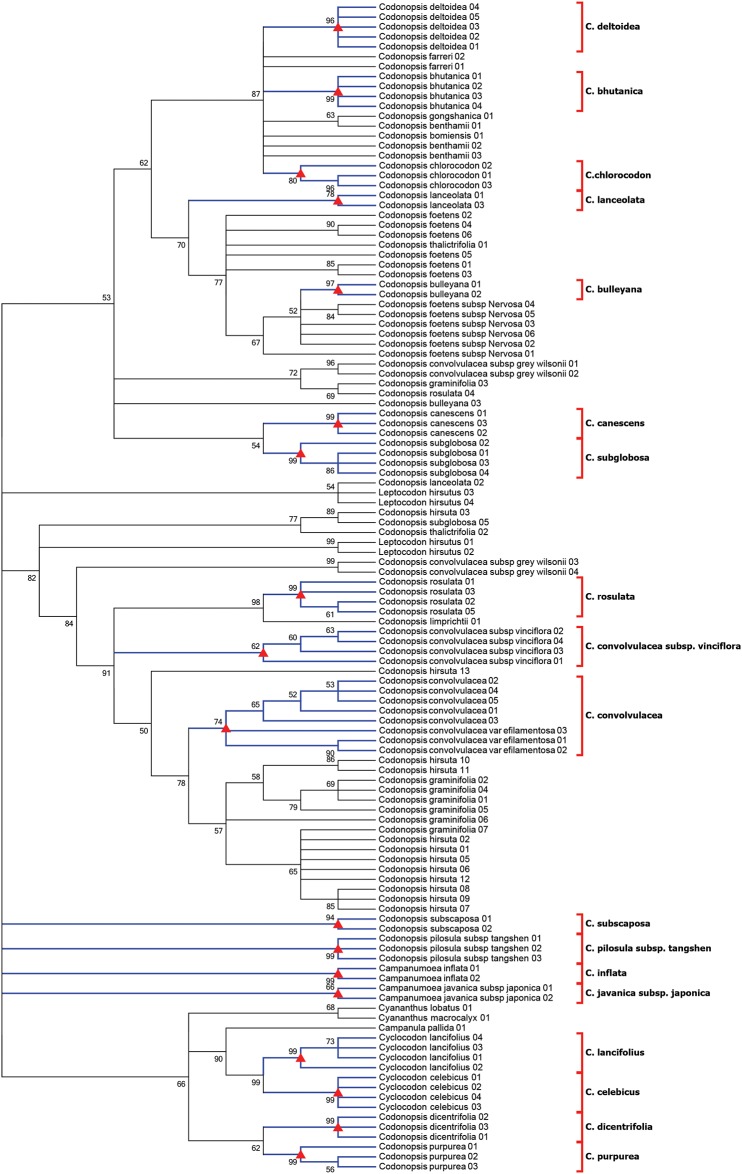
The NJ tree of *Codonopsis* based on the two-locus barcodes 'ITS+*matK*'. Numbers at nodes, bootstrap values with 1000 replicates (only values>50 were shown). Blue species name, resolved species. Black species name, unresolved species.

Specimens of *Codonopsis* are challenging for molecular taxonomy because of their complicated species composition, biparental inheritance of chloroplast, widespread distribution, dynamic evolutionary history, and extensive plastid genome rearrangements during diversification [5, 56, 57]. Our results indicated that the combination of ITS+*matK* performed well with a high resolution for over 80% of species discrimination (Figs 1 and 2). Because the identification success rates of three- or four-loci combinations were lower or slightly higher than the two-locus barcodes, ITS + *matK*, we recommended ITS+*matK* as the most suitable barcodes for large genera.

### Evaluation of combined barcodes for large plant genera

Because of the lower species discrimination rates and varied performance among different plant communities of single barcodes, the combined barcodes have been applied in recent studies [3]. Significant progress has been made in the past decades specifically, the CBOL has advocated *rbcL* + *matK* as the standard combination for combined barcodes [3]. However, several studies have demonstrated that *rbcL* + *matK* have poor identification abilities [58]. Additionally, the two-locus barcodes (ITS + *matK*, ITS + *trnH-psbA*, ITS + *rbcL*, *matK* + *rbcL*, *rbcL* + *trnH-psbA*) and three- or four-loci barcodes (ITS + *matK* + *rbcL*, ITS+ *matK* + *trnH-psbA*, ITS + *matK* + *rbcL* + *trnH-psbA*) have been taken into consideration [3, 20, 22, 23, 27–29, 51, 59–62]. Xu *et al*. [21] have utilized the power of barcodes in the extraordinarily large genus *Dendrobium* based on 1,698 accessions of 184 species, and they found that the combination of ITS *+ matK* performed best in *Dendrobium*, and they also verified the efficiency of ITS *+ matK* in four other large genera including *Ficus*, *Lysimachia*, *Paphiopedilum*, and *Pedicularis*.

**Fig 3:**
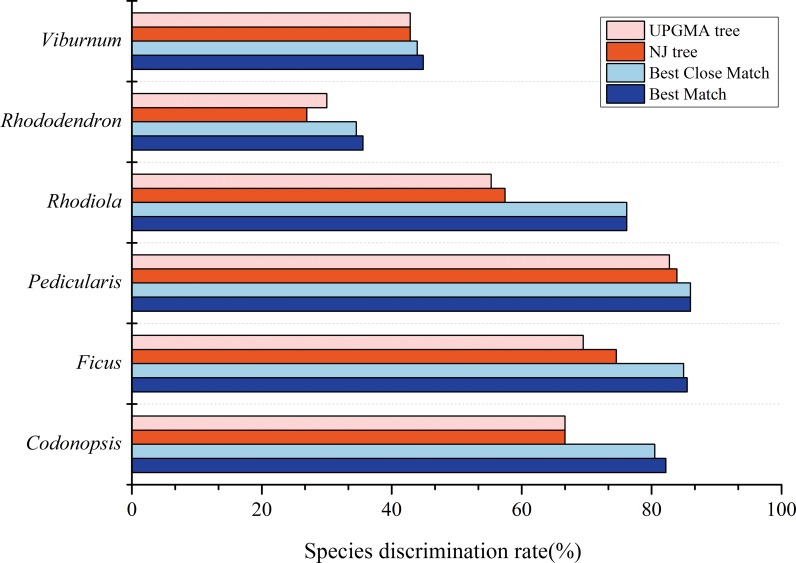
The comparison of discrimination power of ITS + *matK* in large genera using different methods. Four methods ('Best Match, 'Best Close Match', 'NJ tree', and 'UPGMA tree') used to evaluate the discrimination power of 'ITS+matK' in six genera, *Codonopsis*, *Ficus*, *Pedicularis*, *Rhodiola*, *Rhododendron*, and *Viburnum*.

Our results indicated that *rbcL* showed the lowest sequence variation and performed poorly for species identification in the studied genera. Little to no improvement of species resolution was obtained even if *rbcL* was combined with other barcodes (Figure A-F in S2 File, Figure A-Z, a-d in S3 File, Figure A-Z, a-d in S4 File, Figure A-Z, a-d in S5 File, Figure A-Z, a-d in S6 File, Figure A-Z, a-d in S7 File, Figure A-Z, a-d in S8 File). Therefore, combinations with *rbcL* are not suitable for species identification in large genera. In contrast, combinations with *matK* or ITS showed significantly increased in discriminatory power (Fig 1). Specifically, ITS + *matK* performed well in almost all of the studied genera compared to the other single candidates or combinations. Thus, *matK* and ITS are recommended as core barcodes for large genera in our study. Similar results have been found in previous studies [16, 51, 60, 62]. Although other combinations, such as I+T, I+M+R, I+M+T, I+M+R+T also had high discriminatory ability in some genera, however, these barcodes performed more poorly than ITS+*matK* in some of the genera tested (Fig 1). The identification success rates of three- or four-loci combinations were lower or slightly higher than the two-locus barcodes, ITS + *matK*, which suggests that the identification success rates did not increase with an increase in the number of barcodes. Although different methods generated different results, one consistent result was that ITS+*matK* showed better overall performance with multiple evaluation methods (Fig 3, Tables J, K, L, M in S1 File).

## Conclusion

The synthetic analyses of identification ability for all barcodes in the seven species-rich genera (*Codonopsis*, *Dendrobium*, *Ficus*, *Pedicularis*, *Rhodiola*, *Rhododendron*, and *Viburnum*) agreed with previous studies that 'ITS+*matK*' may be the best core barcode combination for large genera in angiosperms [21, 51, 61, 62]. The ITS and *matK* exhibited more variable and informative sites for species identification. The combination of ITS and *matK* performs much better than other single barcode, and was almost equal to the discriminatory power of the three- or four-locus barcodes. Therefore, we propose the combined 'ITS + *matK*' as the core barcode for large plant genera.

## Supporting information

S1 File Supporting tables. Table A. The sampling information of *Codonopsis* used in this study.
Table B. A list of primers used for PCR and sequence in this study.
Table C. The GenBank accession numbers of *Ficus* used in this study.
Table D. The GenBank accession numbers of *Pedicularis* used in this study.
Table E. The GenBank accession numbers of *Rhodiola* used in this study.
Table F. The GenBank accession numbers of *Rhododendron* used in this study.
Table G. The GenBank accession numbers of *Viburnum* used in this study.
Table H. Basic information of the candidate DNA markers in *Codonopsis*.
Table I. Basic information of the candidate DNA markers in *Ficus*, *Pedicularis*, *Rhodiola*, *Rhododendron*, *Viburnum*.
Table J. Summary of the pairwise intra- and inter-specific distances in *Ficus*, *Pedicularis*, *Rhodiola*, *Rhododendron*, *Viburnum*.
Table K. Wilcoxon signed-rank tests of intra- and inter-specific divergence among single barcodes.
Table L. Identification success rates generated by TaxonDNA in the six genera.
Table M. Identification success rates computed by NJ and UPGMA trees in the six genera.
(RAR)

S2 File Barcoding gaps of all genera in this study. (RAR)

S3 File 50% consensus NJ and UPGMA trees based on each barcodes for *Ficus*. Numbers on branches represent NJ or UPGMA support values.
(RAR)

S4 File 50% consensus NJ and UPGMA trees based on each barcodes for *Pedicularis*. Numbers on branches represent NJ or UPGMA support values.
(RAR)

S5 File 50% consensus NJ and UPGMA trees based on each barcodes for *Rhodiola*. Numbers on branches represent NJ or UPGMA support values.
(RAR)

S6 File 50% consensus NJ and UPGMA trees based on each barcodes for *Rhododendron*. Numbers on branches represent NJ or UPGMA support values.
(RAR)

S7 File 50% consensus NJ and UPGMA trees based on each barcodes for *Viburnum*. Numbers on branches represent NJ or UPGMA support values.
(RAR)

S8 File 50% consensus NJ and UPGMA trees based on each barcodes for *Codonopsis*. Numbers on branches represent NJ or UPGMA support values.
(RAR)
